# Do the Manual or Computer-Controlled Flowmeters Generate Similar Isoflurane Concentrations in Tafonius?

**DOI:** 10.3389/fvets.2019.00160

**Published:** 2019-05-29

**Authors:** Mathieu Raillard, Olivier Levionnois, Paul MacFarlane

**Affiliations:** ^1^Bristol Veterinary School, University of Bristol, Bristol, United Kingdom; ^2^Section of Anaesthesiology and Pain Therapy, Department of Clinical Veterinary Medicine, Vetsuisse Faculty, University of Bern, Bern, Switzerland

**Keywords:** anesthesia, flowmeter, horses, Tafonius, TEC 3, vaporizer, ventilator, veterinary

## Abstract

**Introduction:** Tafonius is an anesthesia machine with computer-controlled monitor and ventilator. We compared the isoflurane fluctuations in the circuit with manual (MF) or computer-driven (CF) flowmeters, investigated the origin of the differences and assessed whether isoflurane concentration time course followed a one-compartment model.

**Material and Methods:** A calibrated TEC-3 isoflurane vaporizer was used. Gas composition and flows were measured using a multiparametric monitor and a digital flowmeter. Measurements included: (1) Effects of various F_i_O_2_ with MF/CF on the isoflurane fraction changes in the breathing system during mechanical ventilation of a lung model; wash-in kinetic was fitted to a compartmental model; (2) Gas outflow at the common gas outlet (CGO) with MF/CF at different F_i_O_2_; (3) Isoflurane output of the vaporizer at various dial settings with MF/CF set at different flows without and with reduction of the CGO diameter.

**Results:** (1) The 3% targeted isoflurane concentration was not reached; additional time was required to reach specific concentrations with CF (lowest F_i_O_2_, longer time). The exponential course fitted a two-compartment model; (2) Set and measured flows were identical with MF. With CF at 0.21 F_i_O_2_, flow was intermittently 7.6 L min^−1^ or zero (mean total: 38% of the set flow); with CF at 1.00 F_i_O_2_, flow was 10.6 L min^−1^ or zero (mean: 4–5.3 L min^−1^); with 0.21 < F_i_O_2_ < 1.00, combined flow was intermittent (maximum output: 15.6 L min^−1^); (3) With MF, isoflurane output was matching dial setting at 5 L min^−1^ but was lower at higher flows; with CF generating intermittent flows, isoflurane output was fluctuating. With the 4 mm diameter CGO, isoflurane concentration was close to dial setting with both MF and CF. With a 14 G CGO, isoflurane concentration was lower than dial setting with MF, higher with CF.

**Conclusions and Clinical Relevance:** Using MF or CF led to different isoflurane fraction time course in Tafonius. Flows were lower than set with CF; the TEC-3 did not compensate for high/intermittent flows and pressures; the CGO diameter influenced isoflurane output.

## Introduction

Tafonius (Hallowell EMC and Vetronic Services LTD, UK) is a large animal anesthesia machine with integral computer-controlled monitor and ventilator. Although fresh gas flow (FGF) into the breathing system is conventionally controlled by a manually-driven flowmeter (MF), a computer-driven flowmeter (CF) can be used as an alternative. This feature is particularly useful to administer oxygen-air admixtures in versions of the machine fitted with an oxygen (O_2_) and nitrous oxide (N_2_O) manual flowmeters but no medical air flowmeter. When using the CF, the user sets the targeted inspired oxygen fraction (F_i_O_2_) and the total FGF. In an attempt to deliver the desired gas mixture, oxygen (O_2_) from the pipeline or cylinder supply is blended with room air pumped into the anesthetic machine. The data from the gas measurement module attached to the anesthetic machine is used by the computer to determine the required flows of air and/or O_2_ into the system.

Delivery of inaccurate amounts of volatile agents during equine anesthesia can represent a serious safety concern. Insufficient volatile concentrations might lead to movements or awaking of anesthetized horses; excessive concentrations might result in cardiovascular and respiratory complications, prolonged and poor recoveries. Based on personal experience in anesthetized horses, the authors noticed that using the CF instead of the MF altered the inspired volatile agent fraction reached and on the time required to reach similar fractions. It was hypothetized that, at similar FGF settings, changes in time of volatile agent fraction in the breathing system would be different between MF and CF. The aim of this bench study was to compare the isoflurane fraction fluctuations in the Tafonius anesthetic machine when using the MF or CF and investigate the origin of the differences observed. Wash-in and wash-out kinetics of isoflurane in a breathing system have been described to follow a one-compartment model and are characterized by the time constant (1). The present study also aimed to investigate whether the isoflurane time course followed this assumption with the Tafonius anesthetic machine.

## Materials and Methods

### Equipment

Tafonius 07 (Hallowell EMC and Vetronic Services LTD, UK) was used in this study. Oxygen and N_2_O (but no medical air) manual flowmeters were present on this version of the machine. The machine had been serviced and calibrated the week before the experiment. A recently serviced and calibrated Datex-Ohmeda TEC-3 isoflurane vaporizer was fitted onto the backbar. Prior to each experimental procedure the anesthetic machine was connected to the hospitals piped O_2_ supply and isoflurane added to the vaporizer until the fill gauge was at the recommended maximum level. The anesthetic machine was switched on, the piston zeroed and the automatic leak and compliance check ran following the manufacturer's instructions. The buffer value for the ventilator setting of the Tafonius was set at 15 L. The total volume of the breathing system was calculated to be 28 L (manufacturer's information: breathing tubes [6 L], down-pipes and area above the soda-lime [7 L], and buffer volume [15 L]).

In addition to the monitoring unit of the Tafonius, a Datex-Ohmeda S/5 anesthetic monitor was used throughout the study to measure fractions of O_2_ (F_i_O_2_, ${\text{F}}_{{\text{E}}^{\text{′}}}$O_2_) and isoflurane (F_i_ISO, ${\text{F}}_{{\text{E}}^{\text{′}}}$ISO) within the breathing system (mean sampling rate of 150 mL min^−1^; the extracted volume was not redirected to the breathing system). Before each experiment, this monitor's gas module was calibrated using a calibration gas (Quick Cal Calibration gas, Ref: 755583-HEL [CO_2_ 5.00%, O_2_ 55.0%, N_2_O 33.0%, Desflurane 2.00%] GE Healthcare, Helsinki, Finland) according to the manufacturer's recommendations.

### Phase 1: Effects of Various F_i_O_2_ Settings on the Isoflurane Fraction in the Breathing System During Mechanical Ventilation of a Lung Model

The breathing system was connected to an artificial lung constructed from a rubber reservoir bag (with a volume of 12 L including tubing) within a closed transparent plastic cylinder. This unit was the “bag in bottle” assembly of another large animal ventilator (Dräger Large Animal ventilator; Dräger, UK). Small amount of foam padding was placed within the cylinder to reproduce lung compliance. The artificial lung was connected to the Y-piece of the breathing system. Absence of leak under pressure up to 40 cm H_2_O was checked after assembling the device (manually before connection to Tafonius). Controlled mechanical ventilation was applied (tidal volume: 4 L; respiratory rate: 6 breaths per minute; I:E ratio: 1:3). Peak inspiratory pressure was 35 cm H_2_O. All gas measurements were taken from the gas sampling port of the Y-piece of the breathing system through a three-way tap allowing simultaneous sampling for the monitoring unit of the Tafonius and for the Datex gas module.

#### Step 1a: Effect of F_i_O_2_ and Fresh Gas Flow (FGF) on the Rise (0–3%) of Isoflurane Fraction (${\text{F}}_{{\text{E}}^{\prime }}$ISO)

At the beginning of each step the isoflurane vaporizer was off, and both the breathing system and the artificial lung were pre-filled with the admixture of gases (O_2_ and air) until the ${\text{F}}_{{\text{E}}^{\text{′}}}$O_2_ being tested remained unchanged for 15 min. The vaporizer dial was then turned on 3%, and isoflurane and O_2_ partial pressures measured in the breathing system were manually recorded every minute for 90 min or until ${\text{F}}_{{\text{E}}^{\text{′}}}$ISO remained unchanged for 15 min. The order of experiments was: *(1)* F_i_O_2_ 1.00, MF 5 L min^−1^; *(2)* F_i_O_2_ 1.00, CF 5 L min^−1^; *(3)* F_i_O_2_ 0.21, CF 5 L min^−1^; *(4)* F_i_O_2_ 0.40, CF 5 L min^−1^; *(5)* F_i_O_2_ 0.40, CF 10 L min^−1^; *(6)* F_i_O_2_ 0.70, CF 5 L min^−1^.

#### Step 1b: Effect of F_i_O_2_ on the Reduction (3–1%) of Isoflurane Fraction (${\text{F}}_{{\text{E}}^{\text{′}}}$ISO)

At the beginning of each test the isoflurane vaporizer dial was set on 3%, and both the breathing system and the artificial lung were pre-filled with the admixture of gases (O_2_ and air) until the ${\text{F}}_{{\text{E}}^{\text{′}}}$O_2_ being tested remained unchanged for 15 min. The vaporizer dial was then turned on 1%, and isoflurane and O_2_ partial pressures measured in the breathing system were manually recorded every minute for 90 min or until ${\text{F}}_{{\text{E}}^{\text{′}}}$ISO remained unchanged for 15 min. The order of experiments was: *(1)* F_i_O_2_ 1.00, MF 5 L min^−1^; *(2)* F_i_O_2_ 1.00, CF 5 L min^−1^; *(3)* F_i_O_2_ 0.40, CF 5 L min^−1^; *(4)* F_i_O_2_ 0.70, CF 5 L min^−1^.

For steps 1a and 1b, the difference for the time required to reach a target concentration between MF (as reference) and CF (at different F_i_O_2_) is calculated as a mean of comparison.

#### Step 1c: Comparison of Isoflurane Time Course to One-Compartmental Model

The time course of the isoflurane concentration (${\text{F}}_{{\text{E}}^{\text{′}}}$ISO) during the previous steps was fitted to a pharmacokinetic compartmental model (Phoenix 8.1, Certara USA Inc.), and compared to the ideal behavior of a one-compartmental model for a volume of distribution of 40 L (28 L of the breathing system + 12 L of the lung simulator) and a clearance equal to the input.

#### Step 1d: Effect of F_i_O_2_ Changes on the Stability of the Isoflurane Fraction in the Breathing System Using the CF

The system was filled with O_2_ (F_i_O_2_ 1.0, MF 10 L min^−1^) and isoflurane (set at 3% on the vaporizer) until ${\text{F}}_{{\text{E}}^{\text{′}}}$O_2_ and ${\text{F}}_{{\text{E}}^{\text{′}}}$ISO remained unchanged for 15 min. MF was switched off, CF turned on at 5 L min^−1^, and ${\text{F}}_{{\text{E}}^{\text{′}}}$ISO recorded every minute for 15 min. Afterwards, F_i_O_2_ was decreased stepwise to 0.8, 0.6, and 0.4, and then re-increased to 0.6, 0.8, and 1.00. At each F_i_O_2_, ${\text{F}}_{{\text{E}}^{\text{′}}}$ISO was recorded every minute until the targeted ${\text{F}}_{{\text{E}}^{\text{′}}}$O_2_ was reached and remained unchanged for 15 min, before moving to the next step.

### Phase 2: Measurements of the Gas Outflow at the Common Gas Outlet With MF or CF Set at Different F_i_O_2_

Gas flows were measured with a calibrated portable digital flowmeter (PFM 100 Flow Meter, manufactured by Rusz Instruments Inc. Pittsfield, Massachusetts, USA) connected at the common gas outlet (CGO). Investigated gas (O_2_ or air) was selected on the digital flowmeter to allow accurate measurements.

#### Step 2a: Measurements of the Gas Outflow at the Common Gas Outlet With MF (F_i_O_2_ = 1.0) Across a Range of Flow Settings

The MF was set over a wide range of FGF: 0.5 and 1 L min^−1^ then up to 10 L min^−1^ by 1 L min^−1^ increments.

#### Step 2b: Measurements of the Gas Outflow at the Common Gas Outlet With CF Set at Different F_i_O_2_, Across a Range of Flow Settings

When the CF is used, both the O_2_ flow and the air intake pump (for F_i_O_2_ < 1.0) are intermittent. This intermittent functioning is audible and can be recorded.

The average gas outflow generated by the CF over a range of flow settings (5, 10, 15, and 20 L min^−1^) was calculated at F_i_O_2_ = 1.0 (only O_2_) and F_i_O_2_ = 0.21 (only air). For each setting (FGF, F_i_O_2_), the delivered gas outflow was measured continuously over 2 min, as well as the duration of pump functioning. Combination of these two values provided the average gas outflow (L min^−1^).

### Phase 3: Measurements of the Isoflurane Output of the TEC-3 Vaporizer at Different Dial Settings (0.5–5%), With MF or CF (set at FiO2 of 0.21 or 1) and Across a Range of Flow Settings (5–20 L min^−1^)

The CGO (4 mm. internal diameter) was connected to a 22 mm scavenging corrugated hose.

#### Step 3a:

The scavenging corrugated hose was directed to a F/air canister for waste anesthetic gases (Hanna pharmaceuticals, UK) and the vaporizer dial was successively turned on 0.5-1-1.5-2-2.5-3-3.5-4-4.5-5% and the isoflurane output measured at *(1)* F_i_O_2_ 1.00, MF 5–10 L min^−1^; *(2)* F_i_O_2_ 1.00, CF 5-10-15-20 L min^−1^; *(3)* F_i_O_2_ 0.21, CF 5-10-15-20 L min^−1^. The isoflurane output (${\text{F}}_{{\text{E}}^{\text{′}}}$ISO) was measured by the gas analyser with the sampling line attached to a 20 G needle inserted through the corrugated hose close to the CGO.

#### Step 3b:

An average isoflurane output was measured by collecting the outflow for 4 min within a 30 L rubber bag instead of the absorption canister, and measuring its final isoflurane concentration. This was performed at 5 L min^−1^ with: *(1)* MF, F_i_O_2_ 1.00; *(2)* CF, F_i_O_2_ 1.00; *(3)* CF, F_i_O_2_ 0.21.

These measurements were repeated with a connector reducing internal diameter by introducing and sealing a 14 G needle in the CGO before the corrugated tube.

**Figure 1:**
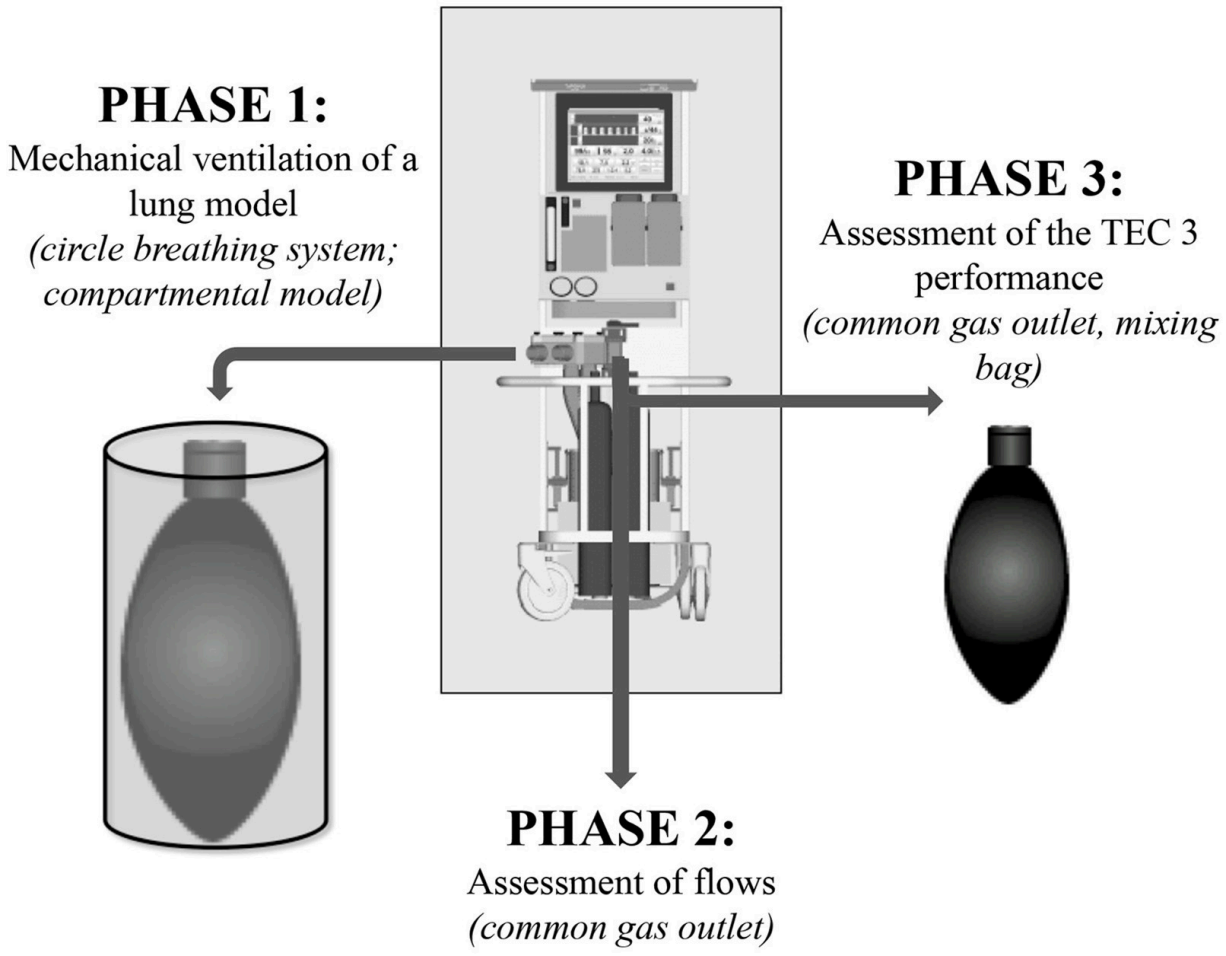
Steps of investigation of the manual or computer-driven flowmeters' different performance on isoflurane changes in Tafonius.

The figure 1 summarizes the different steps of the investigations.

## Statistical Analysis

Each experiment was conducted only once so statistical analysis were not performed. Phoenix 8.1, Certara USA Inc. was used for the pharmacokinetics modeling. For the graphical representations of the additional time required to reach a specific isoflurane concentration at different settings, SigmaPlot for Windows 13.0, Systat Software Inc, CA, USA was used.

## Results

### Phase 1: Effects of Various F_i_O_2_ Settings on the Isoflurane Fraction in the Breathing System During Mechanical Ventilation of a Lung Model

#### Step 1a and Step 1b

**Figure 2:**
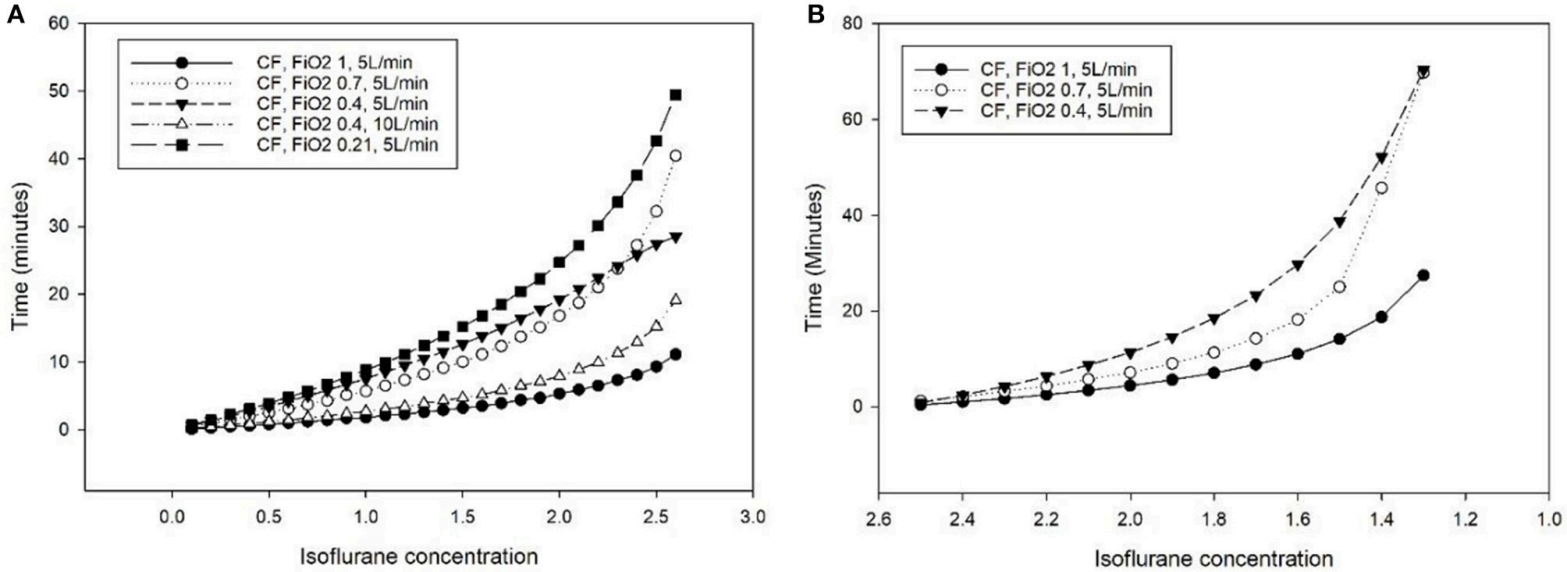
Additional time (minutes) required to reach a specific isoflurane concentration in the breathing system of the Tafonius anesthesia machine during mechanical ventilation of a lung model (bag in a bottle) with a computer-driven flowmeter (CF) at different settings, compared to the use of a manual flowmeter at 5 L minute^−1^. **(A)** Isoflurane increase from 0 to 3% (dial setting of a calibrated TEC 3 vaporizer). **(B)** Isoflurane decrease from 2.7 to 1%.

The time difference between MF and CF (at different F_i_O_2_) in order to reach a target concentration obtained from steps 1a and 1c is presented in Figure 2 (SigmaPlot for Windows 13.0, Systat Software Inc, CA, USA).

#### Step 1c

**Figure 3:**
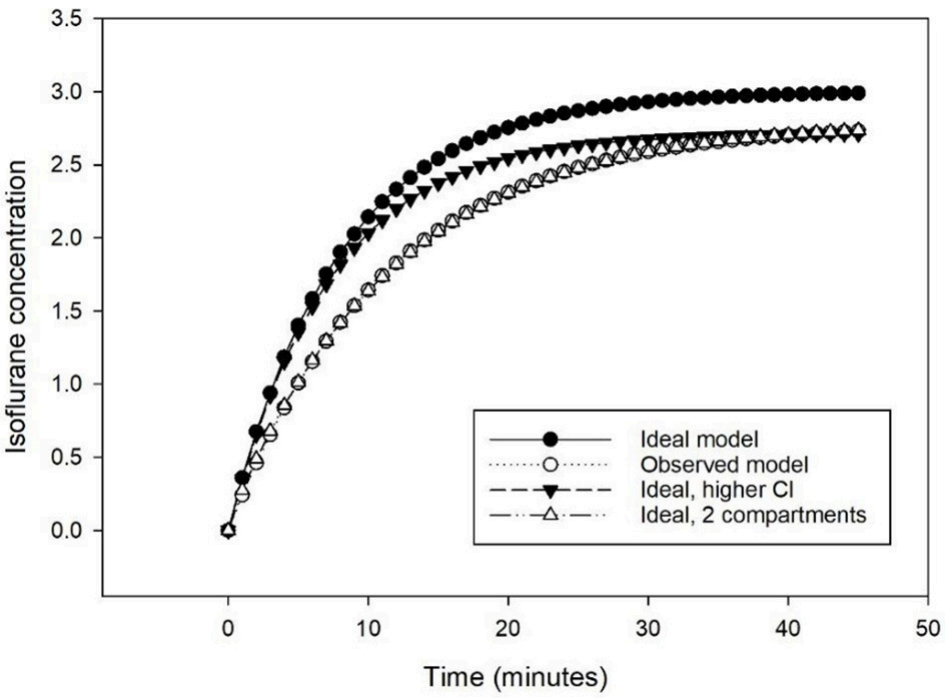
Predicted isoflurane time course to increase from 0 to 3% at 5 L min^−1^ following a one-compartmental model of 40 L and an elimination of 5 L min^−1^ (Ideal model), with an elimination of 5.52 L min^−1^ (Ideal, higher Cl), and with a 2-compartments model of 40 L and an elimination of 5.52 L min^−1^ (Ideal, 2 compartments), compared to the observed time course (Observed model).

The time course obtained with MF differed from the ideal one-compartment model (V_d_ = 40 L, k_e_ = 0.125 min^−1^, τ = 8 min, Cl = 5 L min^−1^). The targeted isoflurane concentration (3%) was not reached requiring a higher elimination constant (k_e_ = 0.138 min^−1^, τ = 7.25 min, Cl = 5.52 L min^−1^). The exponential course fitted better a model including two rather than one compartment (Figure 3).

#### Step 1d

Changes in F_i_O_2_ had no effect on the steady isoflurane partial pressure in the breathing system.

### Phase 2: Measurements of the Gas Outflow at the Common Gas Outlet With MF or CF Set at Different F_i_O_2_

#### Step 2a: Measurements of the Gas Outflow at the Common Gas Outlet With MF (F_i_O_2_ = 1.0) Across a Range of Flow Settings

Set and measured flows were identical when the MF was used.

#### Step 2b: Measurements of the Gas Outflow at the Common Gas Outlet With CF set at Different F_i_O_2_, Across a Range of Flow Settings

With CF at F_i_O_2_ of 0.21 (delivering only air), the air outflow was intermittently 7.6 L min^−1^ or none. Based on recorded durations and frequencies (Table 1), the mean total flow was 38% of the set flow, reaching continuous flow (7.6 L min^−1^) for a set FGF of 20 L min^−1^.

**Table 1:** Calculation of the mean fresh gas flows (FGF) at the common gas outlet of the Tafonius anesthesia machine with the computer-controlled flowmeter (CF) set at different F_i_O_2_ and FGF based on outflow measurements, frequency, and duration of the air or O_2_ intakes.

**F_**i**_O_**2**_**	**FGF (L minute^**−1**^)**	**Number of air/O_**2**_ intakes over 120 s**	**Approximative duration (seconds) of air/O_**2**_ intakes over 120 s**	**Calculated mean FGF (L minute^**−1**^)**
0.21	5	12	30	1.9
	10	12	60	3.8
	15	12	90	5.7
	20	1	120	7.6
1	5	12	45	4.0
	10	10	50	4.4
	15	12	55	4.9
	20	12	60	5.3

With CF at F_i_O_2_ of 1.00 (delivering only O_2_), the O_2_ outflow was intermittently 10.6 L min^−1^ or none. Based on recorded durations and frequencies (Table 1), the mean total flow was between 4 and 5.3 L min^−1^, varying mildly with the set FGF and never reaching continuous flow.

With CF at 0.21 < F_i_O_2_ < 1.00 (mixing air and O_2_), the combined outflow was intermittent with a maximum output of 15.6 L min^−1^.

### Phase 3: Measurements of the Isoflurane Output of the TEC-3 Vaporizer at Different Dial Settings (0.5–5%), With MF or CF (set at F_i_O_2_ of 0.21 or 1.0) and Across a Range of Flow Settings (5 to 20 L min^−1^)

With MF, the isoflurane output of the vaporizer was matching the dial setting at 5 L min^−1^ and 76.6% (± 0.06) of it at 10 L min^−1^.

With CF set at 20 L min^−1^ for F_i_O_2_ of 0.21 (continuous FGF of 7.6 L min^−1^), the isoflurane output of the vaporizer was 87.9% (± 0.03) of it.

For other settings of CF, the intermittent flow generated a fluctuating vaporizer output with a rapid sigmoidal increase up to a peak value, followed by a slower exponential decrease down to a basal value (Table 2), maintained until the next intermittent flow.

**Table 2:** Peak and basal isoflurane concentration (in % of the dialed concentration) generated by the intermittent flow of the computer-driven flowmeter at different F_i_O_2_ and across a range of isoflurane dial setting of the vaporizer.

	**F**_****i****_**O**_****2****_ **1.00**								**F**_****i****_**O**_****2****_ **0.21**	
	**5 L min**^****−1****^		**10 L min**^****−1****^		**15 L min**^****−1****^		**20 L min**^****−1****^		**5 L min**^****−1****^	
**Isoflurane (%)**	**Peak (%)**	**Basal (%)**	**Peak**	**Basal (%)**	**Peak**	**Basal (%)**	**Peak**	**Basal (%)**	**Peak**	**Basal (%)**
0.5	380	116		90		88		90		220
1.0	200	100		86		82		86		140
1.5	147	93		80		80		80		114
2.0	140	95		85		85		90		110
2.5	124	92		84		84		84		104
3.0	114	90		87		87		87		104
3.5	111	89		83		83		86		100
4.0	105	88		83		83		85		98
4.5	96	84		80		80		82		98
5.0	100	82		78		76		78		98

With the 4 mm diameter CGO, the average isoflurane concentration obtained after a 4-min collection in a bag (5 L min^−1^) was close to the dial setting with both MF and CF (Table 3). With the reducing connector to 14 G, the average isoflurane concentration was lower than the dial setting (85%) with MF, and higher than the dial setting with CF (particularly when dialed at 1.00%).

**Table 3:** Mean isoflurane concentration obtained by a 4-min collection in a bag at different dial setting of the isoflurane vaporizer under different settings (Manual flowmeter at F_i_O_2_ 1.00, Computer-controlled flowmeter at F_i_O_2_ 0.21 and 1.00, fresh gas flow set at 5 L min^−1^).

**Flowmeter**	**F_**i**_O_**2**_**	**% isoflurane vaporizer setting**	**% isoflurane measured in collection bag with 14 G**	**% isoflurane measured in collection bag with 4 mm**
MF	1.00	0.50	N/A	0.50
		1.00	0.86	1.00
		3.00	2.50	3.00
CF	0.21	0.50	N/A	0.53
		1.00	2.20	1.00
		3.00	3.30	2.70
	1.0	0.50	N/A	0.70
		1.00	2.50	1.10
		3.00	3.70	3.00

## Discussion

When ventilating a lung model, it was challenging to predict the isoflurane fraction course in Tafonius' breathing system when the CF was used. The wash-in kinetics did not follow the expected one-compartment model and variations in isoflurane fraction were slower with the CF compared to the MF, particularly at lower F_i_O_2_. This difference was attributable to *(1)* the discrepancy between flows set on the CF and actual lower delivered flows and *(2)* to the fact that the isoflurane output of the TEC-3 vaporizer was inaccurate for flows higher than 5–7.5 L min^−1^, for intermittent flow or for flows entering it at high pressures. Interestingly, a smaller tubing downstream the TEC-3 vaporizer worsened the accuracy of isoflurane output.

Factors governing the time course of a change in partial pressure of a volatile anesthetic in a circle breathing system are: *(1)* the volume of the system; *(2)* the FGF and the concentration of anesthetic in the gas admixture entering the system, *(3)* the extent to which circuit components absorb the anesthetic and the extent to which the anesthetic is degraded by the soda lime; *(4)* the uptake of anesthetic by the animal when connected to the breathing system; *(5)* the flow and concentration of anesthetic in the gas admixture leaving the system. The concentration of an anesthetic gas in a breathing system is expected respond to an equation of a simple compartment model equation (2):

$$\begin{array}{l} C\left(t\right)=Css\;\times \;\left(1-e^{-t/\tau }\right) \end{array}$$

where C(t) and Css are the time dependent and steady state concentrations of the volatile agent considered, respectively. A steady state is reached when outflow of gases equals the inflow, in ~3 times the time constant τ (ratio between volume of the breathing system and FGF in case no animal is uptaking the anesthetic gas) (3). However, in the present study, when the volume of the system was forced at 40 L, data fitted better a two-compartment model precluding the use of the time constant to compare the different scenarios.

The 3% targeted isoflurane concentration was not reached in our study. A higher than expected elimination constant was necessary to obtain a good fit of the model with the observed data. This suggests that isoflurane and oxygen were not leaving the system in the same proportion at which they were entering it, isoflurane “elimination” being greater. Three hypotheses could be considered. First, some isoflurane could be degraded by the soda lime (4–7). Second, isoflurane could be absorbed by components of the breathing system, particularly by the rubber bellows of the lung model (8). Rubber is more permeable to anesthetic agents than other components of the breathing system and substantial absorption of isoflurane is likely to happen in clinical anesthesia conditions (8). If this were to be the case, the artificial lung would be the most important site of absorption and the amount of absorbed isoflurane in Tafonius under clinical conditions could be less than what potentially happened in the present study. Third, the dump valve function could be relevant. The dump valve is the equivalent in Tafonius to a pop-off valve in other ventilators and it is computer-controlled. It opens when the level of the piston rises to the point equal to the sum of tidal volume and buffer volume (when the FGF is continuous and greater than patient uptake and leaks, the level of the piston at the end of expiration rises breath by breath). In Tafonius, CGO and exhaust ports are at the same level and close to one another. Since gas movements in a circle breathing system are intermittent (inspiratory and expiratory valves and intermittent ventilation), the mixing of the FGF within the breathing system might not be uniform and a greater portion of the FGF could be scavenged in some circumstances. The fact that our data fitted better a two-compartment model may actually suggest non-uniform mixing of gas inflow in the breathing system. Our study focused on partial pressure of gases at the level of the Y-piece and on flows and vaporizer output at the CGO but waste gases flows and composition were not investigated.

When flows higher than 5 L min^−1^ were delivered through the vaporizer, isoflurane output was lower than dial setting, particularly at settings >2% (Table 2). This finding was in accordance with previous reports (9). The flows delivered when the CF was in use were intermittent and high (7.6 L min^−1^ with 0.21 FiO_2_, 10.6 L min^−1^ with 1.00 FiO_2_ and 15.6 L min^−1^ total combined flow). This could contribute to explain the differences in isoflurane fraction changes between MF and CF.

Although FGF and temperature are known to potentially influence the volatile output in some models of vaporizers (10, 11), the variability in vaporizer output associated with the intermittent flows encountered when CF was used was, not anticipated. A situation that can lead to variable vaporizer output is the “pumping effect” (12). Initially observed during inspiratory phases of intermittent positive pressure ventilation, the “pumping effect” originates from an increase in the resistance in the outlet of the anesthetic machine which leads to an intermittent and variable increase in the anesthetic gas pressure transmitted back to the vaporizer. The gas present in the outlet is saturated with volatile anesthetic; when the backpressure is released, the expanding carrier gas (also saturated) exits both the inlet and the outlet of the vaporizer chamber. The gas leaving the inlet enters the bypass and adds to the vaporizer output, hence the increase of the final vapor output (12). Compared to earlier versions, various modifications have been performed in the Mark 3 to reduce the impact of the pumping effect: the volume of the vaporizing chamber has been reduced in order to minimize the effect of compression. The vapor control channel has been placed on the outlet side of the vaporizing chamber in order to make the resistance of the chamber outlet higher than that of the inlet; a small annular expansion chamber, unprovided with wicks, adjacent to the vaporizing chamber inlet and a long, narrow, annular throat, without wicks, leading down from the expansion chamber to the liquid volatile has been added to confine anesthetic vapor to regions of the chamber remote from the inlet (13). Performance of the Cyprane Fluotec Mark 3 for halothane was evaluated and compared to the TEC 2 and the pumping effect seemed eliminated (13). However, only fairly modest pressures (up to 35 cm H_2_O) were investigated in the latter study. With the CF in Tafonius, O_2_ bypasses the MF and likely reaches the system at higher than atmospheric pressure upstream the vaporizer. This pressure will further increase if the diameter of the tubing is decreased downstream (Haggen Poiseuille equation). Oxygen pressures reaching the vaporizer were not measured but are expected to be markedly above the usual subatmospheric pressure of usual fresh gas inlet. Plenum vaporizer are designed to work at atmospheric pressure and, likely, cannot compensate for high gas pressures. We believe that, under such conditions, an alteration of the splitting ratio in the vaporizer could also explain our findings. Performance of a TEC-3 vaporizer at different given known pressures has not been reported yet and deserves characterization when Tafonius is used in CF mode.

There are several limitations to the present study. Since each experiment was conducted only once, no statistical analysis was performed. Although there is no reason for the results to be markedly different using the same equipment in the same situation, this was not investigated. It could have been interesting to repeat the experiment on several different Tafonius but the ventilator tested was an early model and may not represent more recently constructed units. We used an infrared multi-gas analyser (Datex-Ohmeda S/5 anesthetic monitor) to measure isoflurane tension. The monitor was calibrated prior to each experiment according to the manufacturer's instructions. This is a single point calibration. Gas chromatography (often considered as the most accurate method to measure the concentration of inhaled anesthetic gases) and infrared gas analysis with the monitor we used cannot be used interchangeably as deviations between the techniques exist and performances of individual analysers differ unpredictably (14). Advantages of the infrared monitoring are practicality and limited cost: it is readily available and provides continuous data. Although our isoflurane absolute values may not be perfectly accurate because of the technique we used, we standardized the experiment so that the comparison is valid and we believe the infrared analysis and our results are of clinical relevance.

## Conclusion

Under experimental conditions, using MF or CF led to different isoflurane fraction time course in Tafonius. The wash-in kinetics did not follow a one-compartment model and variations in isoflurane fraction were slower with the CF compared to the MF, particularly at lower F_i_O_2_. Actual delivered flows were lower than set with CF and the TEC-3 did not compensate for high/intermittent flows and pressures. It was therefore challenging to predict the isoflurane fraction course in Tafonius' breathing system when the CF was used. Caution is recommended when using the CF.

## Author Contributions

MR: study design, data collection, data analysis, interpretation, redaction of the paper. OL: mathematical modeling, data analysis, interpretation, redaction of the paper. PM: study design, data collection, data analysis, interpretation, redaction of the paper.

### Conflict of Interest Statement

The authors declare that the research was conducted in the absence of any commercial or financial relationships that could be construed as a potential conflict of interest.
